# 分子印迹聚合物在急性冠脉综合征生物标志物测定传感器中的应用

**DOI:** 10.3724/SP.J.1123.2025.08003

**Published:** 2026-02-08

**Authors:** Yufan ZHANG, Jingwen XIA, Jiayuan ZHU, Maosheng ZENG, Jingyi BAI, Qin XU, Hang GAO

**Affiliations:** 扬州大学化学与材料学院，江苏 扬州 225002; School of Chemistry and Materials，Yangzhou University，Yangzhou 225002，China

**Keywords:** 分子印迹聚合物, 急性冠脉综合征, 生物标志物, 生物传感器, 综述, molecularly imprinted polymers （MIPs）, acute coronary syndrome, biomarker, biosensing, review

## Abstract

心血管疾病（CVDs）的高发病急性冠脉综合征率与死亡率对其早期诊断提出了迫切需求。作为常见且严重的心血管疾病亚型，急性冠脉综合征（ACS）因其高发病率和高病死率，成为临床诊断研究的重点领域。分子印迹聚合物（MIPs）凭借对靶标的高选择性识别能力、优异的化学稳定性及低成本制备等优势，在心血管疾病标志物检测中展现出巨大的应用潜力，为ACS的早期诊断提供了创新的技术路径。本文系统综述了MIPs在ACS诊断中的研究进展：首先聚焦常用的ACS生物标志物及其印迹方法，针对心肌肌钙蛋白（cTnI/cTnT）、肌红蛋白（Mb）及肌酸激酶同工酶（CK-MB）等关键标志物，总结了本体聚合、表面印迹、纳米印迹等技术在提高识别效率方面的优化策略。随后重点探讨了基于MIPs的ACS生物传感器研究，详细介绍了以MIPs为识别单元构建电化学传感器、光学传感器及表面等离子体共振传感器的策略及传感器检测ACS标志物的性能，特别强调了MIPs与微流控芯片的集成在实现微量样本快速检测中的技术优势。研究表明，MIPs在ACS诊断中具有特异性强、抗干扰能力好、检测成本低等显著优势，但当前仍面临一些挑战，如低丰度标志物模板分子获取成本高、传感器批间重复性不足、临床转化商业化进程缓慢等挑战。未来需通过优化MIPs合成策略、开发多模式联用检测技术等途径，进一步提升检测性能，推动其在即时检测（POCT）和个性化医疗中的广泛应用。

## Abstract (en)

Cardiovascular diseases （CVDs） are among the leading cause of global morbidity and mortality. Due to their high prevalence and often asymptomatic progression， there is a pressing need for diagnostic tools that enable the early， accurate， and accessible detection of them. Acute coronary syndrome （ACS）， as a common and severe CVDs with high morbidity and mortality rates， has attracted considerable scientific interest. Various methods have been developed to detect ACS rapidly and accurately. Traditional diagnostic methods relying on antibody-based assays are effective. However， they face significant limitations， including high production costs， poor stability under varying environmental conditions， batch-to-batch variability， and cross-reactivity leading to false positives. These challenges have motivated the search for robust， cost-effective alternatives capable of detecting biomarkers with high sensitivity and specificity. Molecularly imprinted polymers （MIPs） have emerged as a promising alternative solution， offering antibody-like molecular recognition capabilities， superior stability， lower production costs， and resistance to harsh environmental conditions. This review systematically examines the latest advancements in MIP-based sensors for ACS biomarker detection in the last fifteen years， including imprinting strategies for key ACS biomarkers， sensor development and integration， and current challenges along with future perspectives. The first section focuses on the molecular imprinting techniques for essential ACS biomarkers， such as cardiac troponin （cTnI/cTnT）， myoglobin （Myo）， and creatine kinase （CK）. It compares whole-protein imprinting with epitope imprinting， highlighting the advantages of the latter in reducing template costs and enhancing binding specificity. Epitope imprinting using short peptide sequences has demonstrated femtomolar detection limits while overcoming challenges associated with large protein templates， such as structural denaturation and difficult template removal. The review also explores innovative approaches like dummy template imprinting， where structurally similar but cheaper molecules are used to create MIPs for high-cost biomarkers， achieving comparable specificity and sensitivity. The second section discusses the integration of MIPs with advanced biosensing platforms. Electrochemical sensors， using MIP-modified electrodes， have achieved remarkable sensitivity and rapid response times， making them suitable for point-of-care testing （POCT）. Optical sensors， particularly those based on surface-enhanced Raman spectroscopy and surface plasmon resonance， enable label-free， real-time detection with ultra-low detection limits. The review also addresses the integration of MIPs with microfluidic technology， where miniaturized devices facilitate automated， high-throughput biomarker analysis. Examples include paper-based microfluidic sensors that combine capillary action with MIP-SERs tags for multiplexed detection， achieving low detection limits without complex instrumentation. Despite these advancements， the review identifies key challenges hindering widespread clinical adoption of the MIP’s based ACS sensor. Although the sensitivity and specificity of MIPs are impressive， they still lag behind those of monoclonal antibodies in some applications， particularly for low-abundance biomarkers. Reproducibility issues arise from variations in polymerization conditions and template removal efficiency. Commercialization barriers include the lack of standardized production protocols and regulatory frameworks for MIP-based diagnostics. The review proposes several strategic directions to address these limitations. Computational modeling and machine learning could optimize monomer selection and polymerization conditions to enhance MIP’s performance. The development of hybrid systems combining MIPs with nanomaterials may further improve sensitivity and signal transduction. Multidisciplinary collaborations among chemists， engineers， and clinicians will be essential to translate laboratory innovations into commercially viable diagnostic tools. Additionally， the integration of MIPs with artificial intelligence machine learning algorithms could support the development of personalized diagnosis and treatment strategies. These future perspectives are likely to have a significant impact on the early diagnosis and treatment of cardiovascular diseases. In conclusion， MIP-based sensors represent a promising direction in ACS diagnostics， offering a unique combination of affordability， stability， and precision. By addressing current technical and translational challenges， MIP technology has the potential to revolutionize early disease detection， particularly in resource-limited areas. This review not only summarizes a decade of research progress but also provides a plan for future developments that could make personalized， decentralized cardiovascular diagnostics a widespread reality.

《中国心血管健康与疾病报告2024》指出，心血管疾病是严重威胁我国乃至全球居民健康的主要疾病之一^［1］^。急性冠脉综合征（acute coronary syndrome， ACS）作为心血管疾病中最为凶险的类型之一，其病理基础为冠状动脉粥样硬化斑块破裂或糜烂，继发血栓形成，导致心肌缺血甚至坏死^［2］^。由于ACS起病急骤，早期症状隐匿，易被忽视或误诊，因此实现ACS的早期快速诊断对改善患者预后、降低死亡率具有非常重要的意义^［3］^。研究表明，若能在症状出现后2 h内完成相关标志物的检测和临床评估，将显著改善ACS患者的预后情况。然而，传统的检测方法高度依赖抗体，存在诊断试剂成本高、生物分子环境稳定性差（易受温度、湿度等因素影响）等问题，容易导致检测结果出现显著偏差，限制了其在临床中的推广应用。因此，开发新型特异性识别元件成为提高ACS疾病诊断率的重要方向之一。分子印迹聚合物（molecularly imprinted polymers， MIPs）是一种仿生材料，通过模板分子与功能单体定向交联聚合，随后去除模板分子形成特异性识别位点，具有稳定性好、可长期保存及成本低廉等优势^［4］^，为ACS疾病标志物的检测提供了经济、可靠的替代方案^［5-9］^。本团队长期从事分子印迹聚合物的制备研究，已发展出多种通用、高效的分子印迹方法，并将其与功能纳米材料结合，开发了一系列用于环境污染物富集与检测的技术^［10-16］^。相关研究基础能为基于分子印迹聚合物的ACS传感器的印迹设计以及疾病标志物检测提供参考。基于心血管疾病诊断的重要性和紧迫性，本论文系统综述了近15年来MIPs在ACS诊断应用中的研究进展，重点探讨其印迹方法和传感器开发，并对该技术当前面临的灵敏度不足、制备工艺不成熟等挑战提出了解决思路。这些问题的突破将有助于进一步推动MIPs在ACS诊疗中的实际应用。

## 1 分子印迹聚合物中常用的ACS生物标志物

心血管疾病诊断中常用的生物标志物主要包括心肌损伤标志物（心肌肌钙蛋白（cTn）、肌红蛋白（Myo）、肌酸激酶（CK）以及心脏型脂肪酸结合蛋白等）、心肌负荷标志物（B型利钠肽）以及炎症标志物（C-反应蛋白^［17］^以及白细胞介素-6等）^［18，19］^。其中，cTn被国际指南推荐为ACS诊断的“金标准”，具有极高的心肌特异性。cTn在心肌细胞损伤后释放入血的时间窗稳定，不仅对急性心肌梗死具备卓越的诊断准确性，还可通过连续监测辅助评估损伤程度与预后风险，是ACS诊断中不可替代的核心标志物。Myo因其在心肌损伤后1~2 h内即可释放入血，具有超早期升高的特性，因此常作为早期排除心肌梗死的重要工具，尤其适用于胸痛患者的快速初步筛查，有助于显著缩短诊断时间窗。CK及其同工酶CK-MB曾长期作为ACS诊断的经典标志物，虽特异性不及cTn，但适用于资源有限的基层医疗组织，且对评估心肌梗死面积具有一定参考价值，在特定临床情境下仍具有应用意义。基于上述3种标志物在ACS临床诊断与管理中的重要价值及其互补性，本文选择它们作为代表性生物标志物，综述MIPs在ACS诊断传感器中的应用进展。

### 1.1 心肌肌钙蛋白

心肌肌钙蛋白T（cTnT）和心肌肌钙蛋白I（cTnI）是诊断ACS的敏感生物标志物^［20，21］^，其血液浓度变化可反映心肌损伤的程度及病理进程。这两种标志物在症状出现后4~8 h内即可在血清中检测到，其浓度升高可持续达7~10天。其中cTnI因其仅由心肌细胞特异性表达，对心肌组织损伤具有较高的鉴别价值，血清cTnI的快速升高往往提示严重的心肌损伤^［22］^。与cTnI类似，cTnT同样是ACS的特异性生物标志物。该蛋白在生理状态下主要存在于心肌纤维中，外周血中含量极低；当心肌细胞受损时，cTnT被释放入血液循环系统。高敏cTnI检测在诊断ACS方面优于高敏cTnT，尤其是在排除非阻塞性ACS患者方面更加具有优势^［23］^。为实现cTn的高效特异性检测，研究人员选用不同模板分子（如完整的cTn蛋白或者其抗原表位肽段）构建MIPs。Karimian等^［24］^以cTnI为模板分子，以邻苯二胺（*o*-phenylenediamine， *o*-PD）为功能单体，通过电化学聚合法在金电极表面构建了能对cTnI识别的MIP层，其解离常数*K*_d_为2.3×10^-13^ mol/L，结合差分脉冲伏安法（differential pulse voltammetry， DPV）实现了对cTnI的特异性检测。完整的cTn蛋白作为模板能保留其天然构象和表位，有利于准确识别，但cTn蛋白相对分子质量大，在印迹过程中三维结构容易被破坏^［25］^，影响印迹效果；此外，cTn蛋白洗脱困难，结合位点可逆性差，不利于MIPs的重复使用，且成本高，限制了其作为模板分子在分子印迹中的应用。表位（epitope）是蛋白质结构中特定的抗原结合区域，通常由9~15个氨基酸残基组成。与全蛋白印迹相比，表位印迹所采用的模板尺寸更小、易于去除，且结构复杂度较低，有助于提高结合亲和力与选择性^［26］^。表位模板对环境条件（如有机溶剂）的敏感性较低，因此拓宽了功能单体的选择范围。此外，表位肽的合成成本通常低于完整蛋白，因此在分子印迹技术中更具应用优势。图1展示了分别采用全蛋白和表位模板进行分子印迹的示意图^［27］^。Cenci等^［28］^采用表位印迹策略，针对cTnI的3个独特肽段（AK9（AYATEPHAK）、NK11（NITEIADLTQK）、NR11（NIDALSGMEGR））设计了MIPs，并首次将其与基质辅助激光解吸电离飞行时间质谱（MALDI-TOF-MS）联用，开发了一种用于心肌损伤标志物cTnI的超灵敏检测方法，其检出限低至300 fmol/L。Peeters等^［29］^则利用cTnI稳定区的一段氨基酸序列（AA：34~126）作为表位模板，合成分子印迹聚合物纳米颗粒（nanoMIPs），并将其与热传递法（heat transfer method， HTM）相结合，构建了一种快速（40 min）、低样本量（120 μL）、低成本且便携的cTnI检测平台。该平台性能可与高灵敏酶联免疫吸附测定法（enzyme-linked immunosorbent assay，ELISA）相媲美，适用于床旁快速诊断。尽管采用特征肽段作为模板能够有效降低成本，但是其结合位点复杂度相对较低，可能限制其对目标物的特异性识别能力。未来，将多表位印迹策略引入cTn检测领域，有望进一步提高检测的准确性与可靠性。

**图1:**
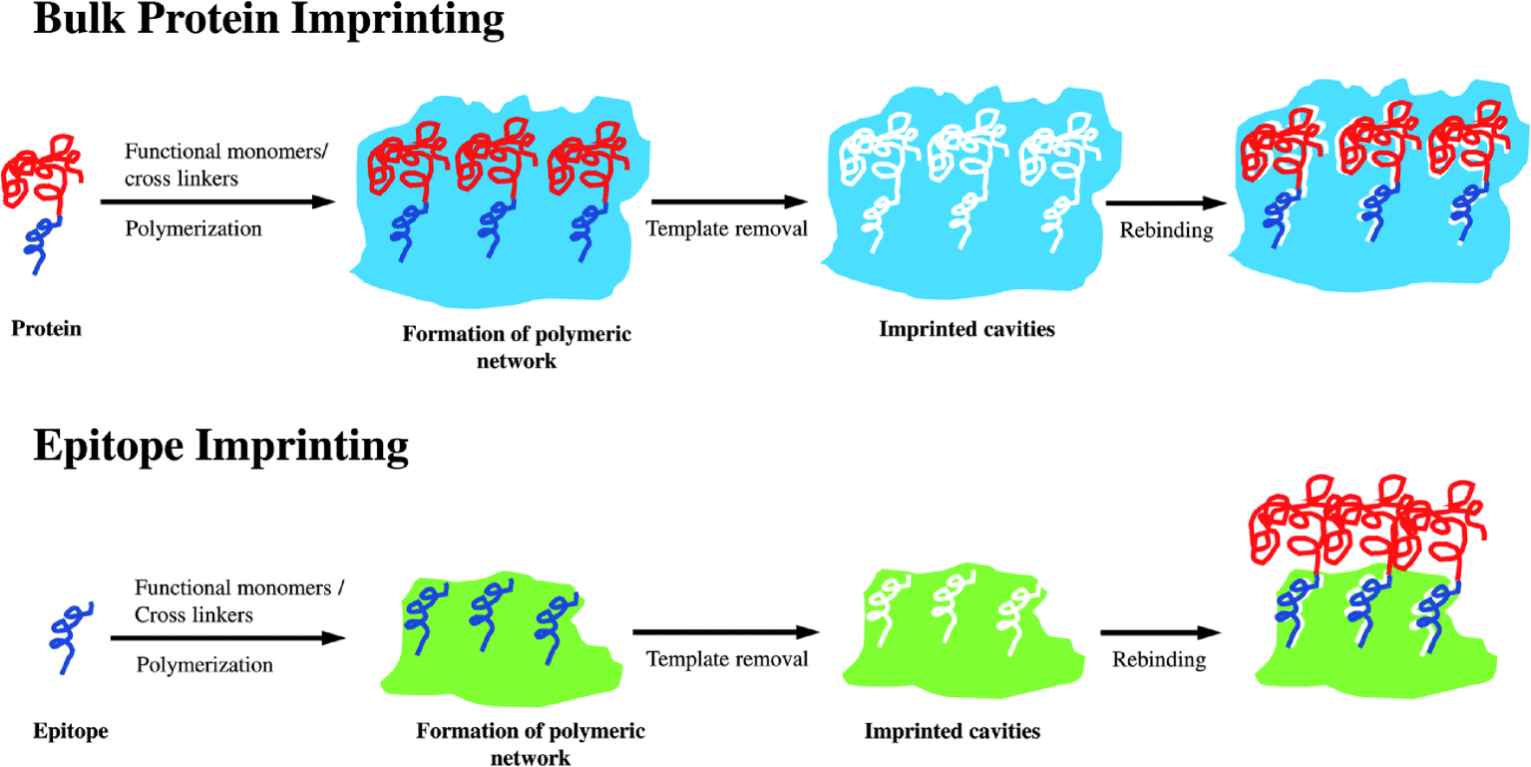
基于整体蛋白及抗原表位的印迹方法^［27］^

### 1.2 肌红蛋白

肌红蛋白是心肌损伤后最早释放至血液中的标志物之一，其释放动力学显著早于cTn^［30］^，可在ACS发病初期提供及时提示。Osman等^［31］^采用微接触印迹技术（μCIP），在表面等离子体共振传感器（surface plasmon resonance sensor，SPR）表面制备了肌红蛋白印迹的聚甲基丙烯-2-羟乙酯-*N*-甲基丙烯酰-L-色氨酸（HEMA-MATrp）共聚物纳米膜。具体过程如下：首先在金片表面依次修饰氨基丙基三乙氧基硅烷（APTES）和戊二醛（GA），以定向固定肌红蛋白模板；随后通过紫外引发聚合反应形成聚合物膜，去除模板后获得具有特异性识别位点的印迹表面。实验结果表明，μCIP制备的单层印迹膜结合位点分布均匀。该传感器对肌红蛋白的检测线性范围为0.1~1.0 μg/mL，检出限达26.3 ng/mL，并已成功应用于急性心肌梗死患者血清中肌红蛋白的检测，表现出灵敏度高、选择性好、操作简便、检出限低以及可实时测量等优势。

Moreira等^［32］^采用模板化电聚合方法，在金丝网印刷电极（Au-SPE）表面制备了聚邻氨基苯酚（PAP）分子印迹膜，构建了一种用于灵敏检测Myo的生物传感器。其检测原理是基于Myo与印迹薄膜特异性结合所引起的电极电化学信号变化进行测定。采用电化学阻抗谱（electrochemical impedance spectroscopy， EIS）测定时，传感器对Myo的线性响应低至4.0 μg/mL，检出限为1.5 μg/mL；而采用方波伏安法（square wave voltammetry， SWV）测定时，线性响应低至3.5 μg/mL，检出限达0.8 μg/mL。该分子印迹薄膜的制备过程如下：首先吸附Myo作为模板，随后电聚合邻氨基苯酚（AP）单体，最后利用蛋白酶K去除模板，从而在聚合物表面形成了与Myo空间结构和电学特性互补的印迹空穴，该印迹膜在水溶液中表现出对Myo良好的识别性能。

Piloto等^［33］^通过自由基聚合法制备了MIP修饰的量子点（MIP-modified quantum dots， MIP-QDs），并基于Myo与MIP-QDs结合引起荧光猝灭的现象，构建了一种用于Myo检测的高灵敏荧光传感器。该传感器在5.06×10^-14^~9.5×10^-11^ mol/L范围内呈现良好的线性响应，检出限低至7.6×10^-15^ mol/L。所制备的MIP-QDs在水溶液中分散性和稳定性良好，即使在复杂血清基质中仍对Myo表现出高选择性和高结合容量，能够有效检测低于心肌梗死临床临界值的Myo浓度。上述研究表明，分子印迹技术可为Myo的高灵敏检测提供新的有效途径。

### 1.3 肌酸激酶

肌酸激酶是一种参与能量代谢的关键二聚体酶，其同工酶（如CK-MB、CK-MM）的检测对ACS等疾病的诊断具有重要意义。在急性心肌梗死发病后3~8 h，CK浓度即显著升高，于10~36 h达到峰值，通常在3~4日恢复至正常水平^［34］^。近年来，基于MIPs的CK检测研究取得显著进展，已从基础的识别界面构建逐步拓展至构象特异性识别层面。Chen等^［35］^以天然CK-MM为模板，通过微热量测定分析筛选出最优聚合体系，采用聚乙二醇-400-二甲基丙烯酸酯（PEG400DMA）作为交联剂、甲基丙烯酸（MAA）作为功能单体，利用微接触印迹法制备出对CK-MM具有高亲和力的MIPs。通过酶联免疫吸附测定法对吸附蛋白进行定量分析，结果显示该MIP对CK-MM的最大结合容量为（2.05±0.07）×10^-10^ mol/cm²，且在复杂人血清基质中仍保持对目标蛋白的特异性识别。进一步研究发现，热变性后的CK-MM因二级结构显著破坏，其与MIPs的结合能力明显降低。该结果证实了MIPs对蛋白质三维构象具有特异性识别能力，为发展基于构象识别的分子印迹技术提供了重要依据。

Tai等^［36］^进一步提出人工抗原表位印迹策略，以包含CK-MB的M亚基369~381序列在内的9种线性表位作为模板，在石英晶体微天平（QCM）芯片表面构建了高选择性MIPs传感界面。研究结果显示，该传感器能够实现对CK-MB、CK-BB和CK-MM 3种同工酶的精准识别，其中对CK-MB检测的线性范围为1~10 ng/mL，检出限达0.5 ng/mL。值得注意的是，亚基构象分析表明，在聚合过程中CK-MB异源二聚体的N端区域发生了特异性构象变化，而C端结构域则保持相对稳定。该研究结果不仅验证了抗原表位印迹技术在蛋白质特异性识别中的可行性，也为发展不依赖抗体的生物传感技术提供了新路径。

Wang团队等^［37］^则聚焦变性蛋白识别，以十二烷基硫酸钠（SDS）处理的变性CK-MM为模板分子，通过热分析优化聚合体系，筛选出以PEG400DMA为交联剂、甲基丙烯酸甲酯（MMA）为功能单体的最佳组合。研究采用ELISA方法检测MIP对目标蛋白（变性CK-MM）的特异性结合，以评估印迹效率；同时结合荧光（fluorescence， FL）检测技术分析非目标蛋白（荧光标记的人血清白蛋白（HSA）与免疫球蛋白G（IgG））的结合量，进一步验证MIP对目标蛋白的高选择性。结果表明，所制备的MIP膜对变性CK-MM表现出优异的识别性能，其印迹因子为8.66，解离常数为3.25×10^-8^ mol/L。在竞争性实验中，MIP对变性CK-MM/变性HSA和变性CK-MM/变性IgG的选择性分别为96.8%和98.7%，证明该材料不仅能识别蛋白质的一级结构特征，还可有效区分由变性引起的二级结构差异，这一研究为复杂生物基质中蛋白质的精准检测提供了新的技术思路。

从早期对天然蛋白的基本印迹，到表位筛选与构象特异性识别技术的突破，再到在变性蛋白及复杂基质中的应用，MIPs在CK检测技术中的发展，为ACS生物标志物的检测提供了多元化的创新策略。

## 2 基于MIPs的ACS诊断传感器研究进展

MIPs凭借其高特异性分子识别能力，可显著提升传感器的选择性和灵敏度^［38-40］^。根据信号转导机制的不同，基于MIPs的ACS标志物传感器主要分为以下3类。

### 2.1 电化学传感器

电化学传感器凭借其高灵敏度、低成本、操作简便及易于微型化等优势^［41-43］^，在心肌标志物即时检测（point of care testing， POCT）领域具有重要的应用价值。其工作原理是通过监测目标分子与电极界面相互作用所引起的电化学信号变化（如电流、阻抗或电位），从而实现对生物标志物的快速定量分析^［44-46］^。例如，Zuo等^［47］^报道了基于MIP的cTnI电化学传感器。他们以cTnI为模板分子，邻氨基苯酚作为功能单体，采用电化学聚合法构建了MIPs修饰的电化学传感器，该传感器对0.05~5.00 nmol/L浓度范围内的cTnI呈现良好的线性响应，检出限为0.027 nmol/L，响应时间小于5 min，并且在血清样本中表现出优异的选择性。

Ma等^［48］^构建了一种集成石墨烯纳米片、多壁碳纳米管、壳聚糖和戊二醛复合材料的电化学传感界面。该界面具有较大的活性比表面积，可有效促进电子传递速率。在此基础上，他们以甲基丙烯酸为功能单体、cTnI为模板分子，在偶氮二异丁腈的引发下，制备了基于多壁碳纳米管/石墨烯纳米片的MIPs传感器。此传感器对cTnI表现出良好的分析性能。He等^［49］^进一步开发了一种基于MIP的电化学发光（electrochemiluminescence， ECL）免疫传感器，实现了cTnI和肌红蛋白（Mb）的同时灵敏检测。该方法通过电聚合多巴胺在电极表面形成分子印迹聚合物层，用以捕获cTnI；随后引入经抗cTnI抗体功能化的ECL探针，以实现信号检测。

Moreira等^［50］^以Myo为模板，开发了一种基于仿生材料的电化学生物传感器。该工作首先在丝网印刷金电极（Au-SPE）的金工作区涂覆羧基化聚氯乙烯（PVC-COOH）膜，利用1-乙基-3-（3-二甲基氨基丙基）碳二亚胺（EDC）和*N*-羟基琥珀酰亚胺（NHS）活化羧基，共价固定Myo，随后以丙烯酰胺为功能单体、*N*，*N*′-亚甲基双丙烯酰胺（NNMBA）为交联剂，在过硫酸铵（APS）引发下进行聚合，形成表面分子印迹层；最后采用草酸溶液去除模板分子。研究者通过傅里叶变换红外光谱和循环伏安法对表面修饰过程进行了验证，电化学阻抗谱和方波伏安法用于性能评价。结果表明，该传感器的阻抗信号与Myo在0.85~4.26 μg/mL范围内呈现良好的线性响应，检出限为2.25 μg/mL，SWV电流信号与Myo质量浓度在1.1~2.98 μg/mL范围内也表现出线性关系。TnT、牛血清白蛋白（BSA）和CK-MB对该传感器的干扰误差分别为7%、11%、2%，表明其具有良好的选择性，适用于生物体中Myo的快速筛查。

此外，为降低MIPs传感器的构建成本并拓宽模板单体的可选范围，Sadeghi等^［51］^提出以细胞色素c（Cyt c）作为虚拟模板，替代价格昂贵且不易纯化的cTnI。他们借助分子对接和分子动力学模拟，优化了功能单体邻苯二胺与模板及目标蛋白之间的相互作用位点及结合能，进而通过电化学聚合构建了MIPs识别界面。实验采用二茂铁甲酸作为竞争性氧化还原探针，结合示差脉冲伏安法，实现了对cTnI的超灵敏检测，其检出限可达10^-14^ mol/L。该策略不仅为高成本或难获取模板分子的替代提供了新思路，也验证了计算辅助方法在MIPs开发中的可行性与有效性。

### 2.2 光学传感器

光学传感器能基于光信号的变化（如强度、波长或偏振态）进行非侵入式检测，具有抗干扰能力强、可实时检测等优点，尤其适用于复杂生物样品中痕量标志物的分析^［52］^。

#### 2.2.1 表面增强拉曼散射（SERS）传感器

SERS技术利用金、银等贵金属纳米颗粒的局域表面等离子体共振效应，可将分子的拉曼信号增强10^6^~10^14^倍，具备快速、高灵敏、特异性强及分子指纹识别等优势^［53］^。Bai等^［54］^开发了一种基于Au@Ag双金属核壳纳米颗粒的MIPs-SERS横向流动试纸条，该传感器利用Au@Ag核壳结构产生的强电磁场增强效应，结合MIPs对cTnI的特异性识别能力，可在15 min内完成对cTnI的检测，检出限低至0.09 ng/mL，并借助拉曼特征指纹峰有效排除了Myo、CK-MB等共存蛋白的交叉干扰。

#### 2.2.2 表面等离子体共振（SPR）传感器

SPR是一种利用光在不同介质中产生消逝波后与等离子波产生共振构建的无标记、超灵敏分析技术^［55］^。当目标物与固定在金属表面的探针分子结合后，会引起金属表面折射率的变化，从而改变共振条件；通过检测反射光强度或共振角位移等参数，即可实现对目标物的高灵敏检测^［56］^。Palladino等^［57］^以cTnT及其4种衍生肽（涵盖N-末端、C-末端及两个抗体识别表位对应的肽段）作为模板分子，以多巴胺作为功能单体，采用多巴胺原位聚合法在金芯片表面构建了cTnT分子印迹层。该传感器对cTnT表现出良好的结合选择性，检出限达到15.4 nmol/L。

Baldoneschi团队^［58］^以去甲肾上腺素（NE）为功能单体制备了MIPs，用于检测cTnI。由于NE比多巴胺多一个羟基，所合成的NE聚合物（PNE）具有更强的亲水性，有助于减少蛋白质的非特异性吸附。该研究团队以cTnI的C-末端（197~210）和N-末端（28~40）肽段为模板分子，在碱性条件下与NE聚合，成功制备了PNE-MIPs。与多巴胺基MIPs相比，NE基MIPs展现出更强的抗干扰能力。基于该材料的SPR传感器对cTnI的检出限低至4.4 nmol/L。研究人员进一步利用碱性磷酸酶标记的抗TnC抗体与MIPs构建夹心结构传感器，该传感器在磷酸缓冲溶液（PBS）缓冲液和人体血浆中均表现出良好的线性和重现性，但检出限存在差异。与未标记TnC抗体相比，使用碱性磷酸酶标记抗体后，PBS中的检出限从（460±56） pmol/L降低至（193±30） pmol/L，血浆中的检出限从（8.9±1.9） nmol/L降至（7.1±0.6） nmol/L。结果表明，以NE为功能单体制备的MIPs能够有效提升传感器的检测灵敏度。

#### 2.2.3 荧光传感器

Piloto等^［59］^开发了一种基于分子印迹技术的新型荧光纤维素膜传感器，用于临床检测Myo。在该传感器的结构中，MIPs作为生物识别元件，量子点则作为荧光探针用于信号检测。当肌红蛋白浓度升高时，传感器膜的荧光强度发生猝灭，在稀释1 000倍的人血清中，该传感器对Myo的检测线性范围为7.39~291.3 pg/mL。

### 2.3 微流控芯片集成传感器

微流控芯片集成传感器结合MIPs的高选择性以及微尺度反应动力学优势，能够实现对ACS标志物的自动化、高灵敏度检测。该技术显著缩短了分析时间，提高了检测效率，在便携式即时诊断中展现出小型化、高通量和实时监测的应用潜力，契合POCT的需求，为心血管疾病的早期筛查与精准诊疗提供了高效解决方案^［60］^。图2展示了微流控芯片传感器的结构示意图及其在心脏标志物无标记检测中的电化学传感过程。Ramakrishna等^［61］^研究了微流控电化学生物传感器在心血管疾病诊断中的应用，重点探讨了其在cTnI与cTnT无标记检测方面的技术进展。

**图2:**
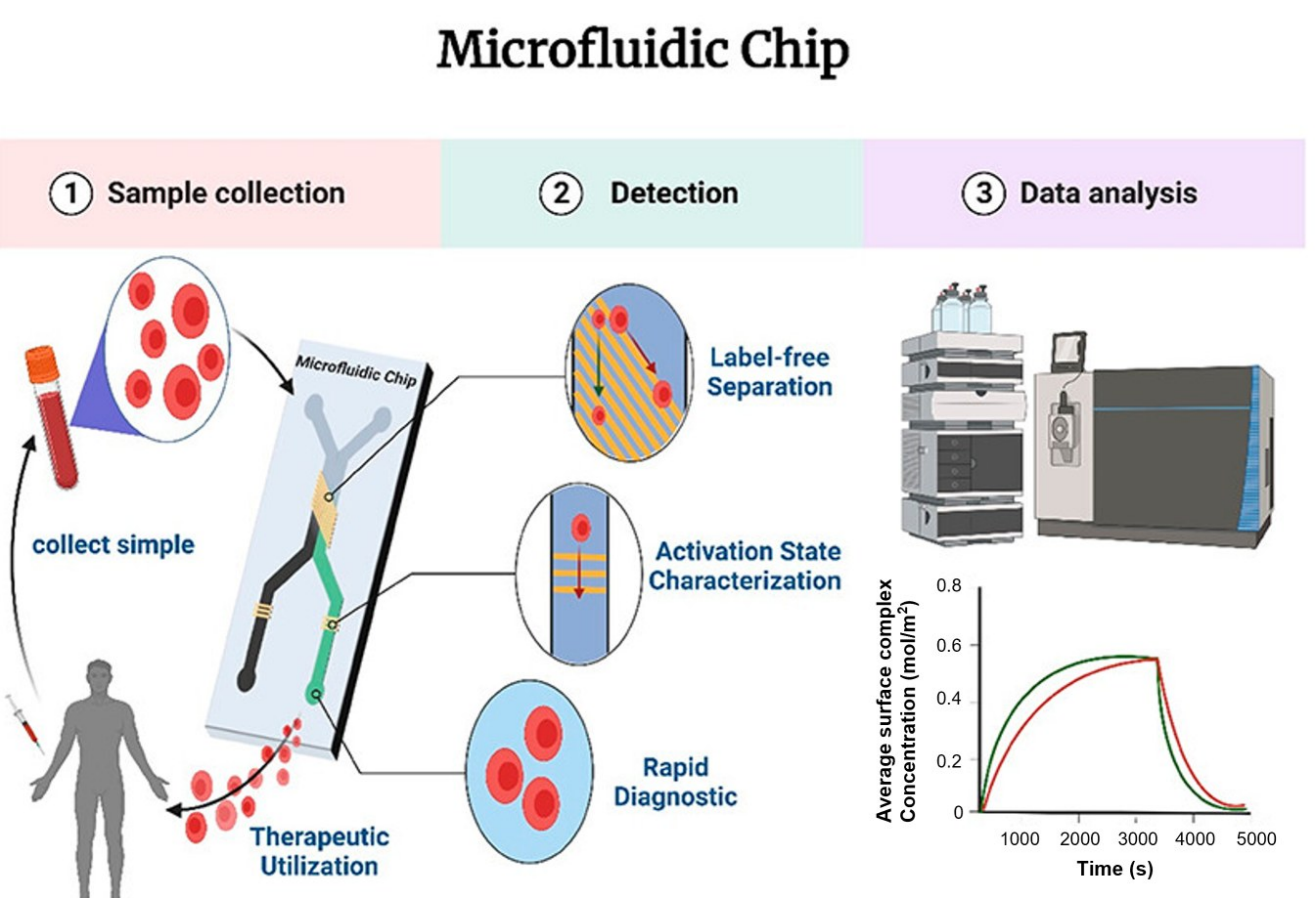
利用微流控电化学生物传感器对心肌生物标志物进行无标记检测的示意图^［61］^

纸基微流控是微流控技术的一个重要分支，其利用纸张的多孔结构和毛细作用实现液体自发驱动，无需依赖外部泵浦系统。基于纸基材料制备的微流控芯片具有成本低、易加工、环境友好等优点^［62，63］^。Lim团队^［64］^采用光刻和蚀刻等微加工技术，开发了一种纸基微流控装置（图3）。结合SERS技术，实现了对cTnT、Myo和CK-MB等多种心肌标志物的同时检测，检出限低至1 pg/mL，且无需复杂校准步骤，为急性心肌梗死等疾病的快速诊断提供了便捷高效的解决方案。Khachornsakkul等^［65］^报道了一种集成MIPs的纸基微流控分析装置，用于检测水、食品及体液中的全氟辛烷磺酸，具备操作简便、响应快速等优势。由于MIPs可以微/纳米颗粒、薄膜等多种形式存在，并具有良好的理化稳定性，通过调控聚合方式可将其灵活集成于微流控系统中，从而提升分析效率，实现高通量检测，展现出在ACS诊断中的应用潜力^［66］^。Li等^［67］^开发了一种旋转式微流控电化学纸基分析装置（MIP-ePADs），将表面分子印迹技术与旋转微流控平台相结合，简化了电化学聚合印迹和模板洗脱流程。并以MIPs作为识别元件，结合电化学检测技术，实现了心肌标志物的高灵敏度检测。

**图3:**
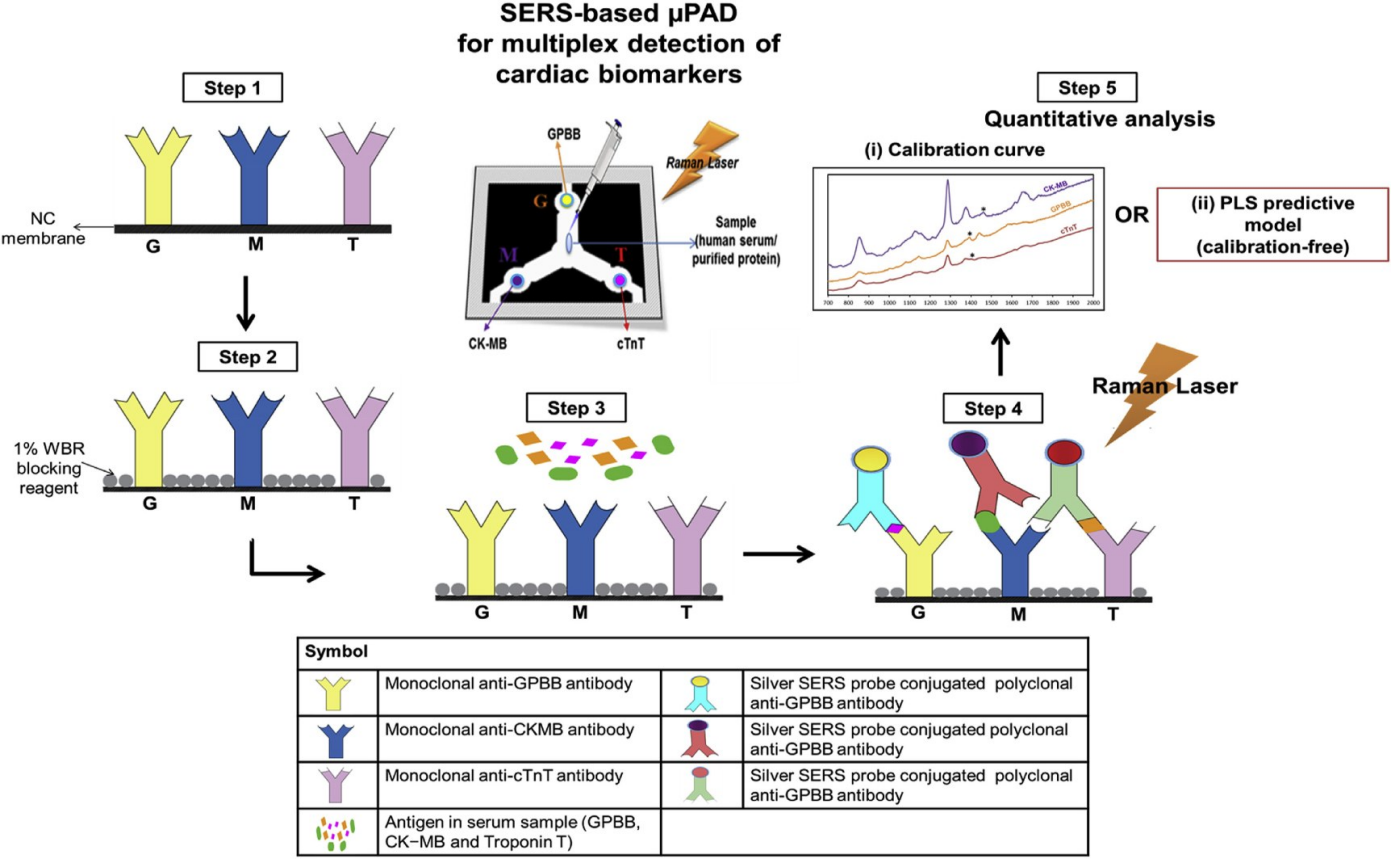
μPAD上各反应区多重SERS检测心肌生物标志物示意图：G区对应GPBB，M区对应CK-MB，T区对应cTnT^［64］^

上述3种传感器在基于MIPs的ACS标志物检测中各有侧重：电化学传感器灵敏度高、检出限低，成本低廉且兼容性好，易于微型化和集成，适用于开发床边检测系统，但其多指标检测能力较弱，常需要前处理步骤以提高选择性，稳定性和重现性也有待提升。光学传感器涵盖荧光、电化学发光及表面等离子体共振等多种模式，具备时空分辨能力，适用于多组分检测，然而通常仪器成本较高，且不便于长期或动态监测。微流控集成传感器样品消耗少，集成度和自动化程度高，能够融合电化学与光学传感单元，实现多指标同步检测，是临床快速诊断的重要发展方向，但其设计与制造工艺较为复杂。

为便于评估不同传感器在临床应用中的潜力，本文调研了上述3种标志物的临床阈值：肌钙蛋白（如cTnI）的阈值通常为0.4 ng/mL，超过该值并结合临床症状可提示心肌损伤^［68］^； Myo的阈值一般为87 ng/mL，其在ACS发病后1~2 h即可升高，常用于早期排除诊断^［69］^； CK-MB的临床阈值约为5 ng/mL，其升高幅度与心肌梗死面积具有一定相关性^［70］^。表1系统总结了基于MIPs的生物传感器在ACS生物标志物（肌钙蛋白、肌红蛋白以及肌酸激酶）检测方面的最新研究进展及线性范围。通过对比ACS的临床阈值与传感器的线性下限，可更清晰地判断各类传感器的检测性能是否符合临床需求。

**表1:** 分子印迹聚合物在急性心肌梗死心肌生物标志物检测中的应用

ACS marker	Template molecule	Functional monomer	Imprinting method	Detection methods	Linear ranges	Ref.
cTnI	cTnI	OAP	electropolymerization	DPV	0.0500-5.00 nmol/L	［^47^］
	AYATEPHAK， NITEIADLTQK，NIDALSGMEGR	IA	photopolymerization	MALDI-TOF-MS	N.D.	［^27^］
	Pseudotemplate of Cytochrome c	*o*-PD	electropolymerization	DPV	4.20×10^-14^-3.00×10^-10 ^mol/L	［^51^］
	CISASRKLQLK	2-AP	electropolymerization	SWV	1.00-10.0 pg/mL	［^71^］
	ALSGMEGRKKKFES	AAM， MAA， MBA， NIPAAm	free radical initiated polymerization	EC-colorimetry	EC：1.00×10^-2^-1.00×10 ^3^ ng/mL； colorimetry：1.00×10^-2^-1.00×10^3^ ng/mL	［^72^］
	ISASRKLQLK	NIPAAm， MBA， APM， TBAm， MAE， RB-TC	free radical initiated precipitation polymerization	SPR	0.780-50.0 ng/mL	［^73^］
	ALSGMEGRKKKFES	MAA， DMAm	free radical initiated polymerization	SERS	0.001-100 ng/mL	［^74^］
Myo	Myo	AAM	bulk free radical polymerization	FL	0.304-571 pg/mL	［^33^］
	Myo	AAM	free radical in-situ polymerization	EIS， SWV	EIS：0.852-4.26 μg/mL； SWV：1.10-2.98 μg/mL	［^50^］
	Myo	AP	electropolymerization	EIS， SWV	EIS：4.00-53.3 μg/mL； SWV：0.0530-53.3 μg/mL	［^32^］
CK	natural CK-MM	MAA	free radical initiated polymerization	ELISA	0.350-3.50 μmol/L	［^35^］
	SDS， denatured CK-MM	MMA	free radical initiated polymerization	ELISA， FL	0.350-3.50 μmol/L	［^37^］

OAP： *o*-aminophenol； IA： itaconic acid； AAM： acrylamide； MBA： *N，N′*-methylenebisacrylamide； NIPAAm： *N*-isopropylacrylamide； APM： *N*-（3-aminopropyl）methacrylamide hydrochloride； TBAm： *N*-*tert*-butylacrylamide； MAE： methacryloyloxyethyl； RB-TC： rhodamine B thiocarbamoyl； DMAm： dimethylaminoethyl methacrylate； SPR： surface plasmon resonance； FL： fluorescence spectroscopy； EIS： electrochemical impedance spectroscopy； N.D.： not detected.

## 3 结果与展望

MIPs能够模拟生物抗体识别目标分子，具备预定性、识别特异性与良好的实用性等特点，且制备过程简单、成本较低，适合大规模生产与推广，在ACS疾病诊断中展现出独特优势。然而，该技术目前仍面临多项挑战：在检测极微量心肌标志物时，需进一步提升灵敏度、改善信噪比并降低检出限；在复杂样本基质中实现生物标志物分子的高特异性识别仍较为困难，需通过优化MIPs设计与合成过程以减少交叉反应；此外，心肌标志物作为模板分子往往含量低、价格昂贵，限制了MIPs的制备效率与经济性；当前MIPs技术的商业化进程也较为缓慢，实际产品少，且缺乏统一的质量控制标准与市场推广体系。未来，推动MIPs技术的发展需从多方面着手：一方面应优化制备工艺与材料配方，以提升性能并降低成本；另一方面需将MIPs与电化学、光学等快速检测技术相结合，构建集成样品处理与检测功能的生物传感平台，从而实现ACS的快速、准确与实时诊断，助力其临床转化。此外，将MIPs传感器与人工智能（AI）和机器学习算法相融合，有望推动个性化诊断与治疗策略的发展。例如，可借助机器学习优化印迹模板和功能单体的筛选过程，利用深度学习构建MIPs性能预测模型，促进AI驱动的MIPs传感器向智能化方向发展，并拓展AI辅助的临床应用场景。这些发展方向有望对ACS的早期诊断和治疗策略产生重要影响，进而提升患者治疗效果、降低死亡率。总之，本综述系统梳理了MIPs在ACS传感器领域的现有进展，并对其未来发展方向与面临的挑战进行了展望。MIPs与传感技术的协同融合，为ACS及其他疾病诊断传感器的研发提供了新思路，也将进一步推动医疗诊断技术的进步。

## References

[1] National Center for Cardiovascular Diseases， The Writing Committee of the Report on Cardiovascular Health and Diseases in China . Chinese Circulation Magazine， 2025， 40（6）： 521 10.3967/bes2024.162 39401992

[2] Boeddinghaus J， Bularga A， Taggart C， et al . Eur Heart J Acute Cardiovasc Care， 2025， 14（3）： 131 39824208 10.1093/ehjacc/zuaf002 PMC11929527

[3] Campu A， Muresan I， Craciun A-M， et al . Int J Mol Sci， 2022， 23（14）： 7728 35887073 10.3390/ijms23147728 PMC9318943

[4] Wang Y， Li N . Chemical Industry and Engineering Progress， 2010， 29（12）： 2315

[5] Sarvutiene J， Prentice U， Ramanavicius S， et al . Biotechnol Adv， 2024， 71： 108318 38266935 10.1016/j.biotechadv.2024.108318

[6] Pilvenyte G， Ratautaite V， Boguzaite R， et al . J Pharm Biomed Anal， 2023， 228： 115343 36934618 10.1016/j.jpba.2023.115343

[7] Çimen D， Bereli N， Günaydin S， et al . Talanta， 2022， 246： 123484 35462248 10.1016/j.talanta.2022.123484

[8] Ouyang M， Tu D， Tong L， et al . Biosens Bioelectron， 2021， 171： 112621 33120234 10.1016/j.bios.2020.112621

[9] Lin X L， Lu M L， Tang R， et al . Guangzhou Chemical Industry， 2024， 52（20）： 1

[10] Shen Y Z， Feng J Z， Wang Z， et al . Anal Chem， 2025， 97（25）： 13487 40546225 10.1021/acs.analchem.5c01915

[11] Wang Z， Shen Y Z， Xu M， et al . Sens Actuators B Chem， 2024， 414： 135924

[12] Chen L， Song M X， Guan J， et al . Talanta， 2021， 225： 122050 33592772 10.1016/j.talanta.2020.122050

[13] Yang X Y， Chen L， Xiong X X， et al . Sens Actuators B Chem， 2020， 304： 127321

[14] Yang X Y， Gao Y， Ji Z P， et al . Anal Chem， 2019， 91（15）： 9356 31313578 10.1021/acs.analchem.9b01739

[15] Wang J， Xu Q， Xia W W， et al . Sens Actuators B Chem， 2018， 271： 215

[16] Wang H M， Xu Q， Wang J， et al . Biosens Bioelectron， 2018， 100： 105 28881228 10.1016/j.bios.2017.08.063

[17] Lee M H， Liu K H， Thomas J L， et al . Biosens Bioelectron， 2022， 200： 113930 34979348 10.1016/j.bios.2021.113930

[18] Chen K-J， Jiang Y-R . Chinese Journal of Integrated Traditional and Western Medicine， 2009， 29（7）： 581 19852283

[19] Zhu Y J， Deng X J . Laboratory Medicine and Clinic， 2025， 22（9）： 1199

[20] Xu Z Y， Gao G W， Li Y S， et al . Progress in Chemistry， 2023， 35（8）： 1266

[21] Hatir P C， Marinangeli A， Bossi A M， et al . Talanta Open， 2025， 11： 100439

[22] Li Y X， Luo L X， Kong Y Q， et al . Biosens Bioelectron， 2024， 249： 116018 38232451 10.1016/j.bios.2024.116018

[23] Tveit S H， Myhre P L， Hanssen T A， et al . Sci Rep， 2022， 12（1）： 945 35042885 10.1038/s41598-022-04850-7 PMC8766564

[24] Karimian N， Turner A P F， Tiwari A . Biosens Bioelectron， 2014， 59： 160 24727601 10.1016/j.bios.2014.03.013

[25] Qin H， Han J Z . Food Science， 2007， 28（8）： 577

[26] Ma Y， Yao Z Y， Zhang Y， et al . Science China： Life Sciences， 2025， 55（3）： 544

[27] Khumsap T， Corpuz A， Nguyen L T . RSC Adv， 2021， 11（19）： 11403 35423617 10.1039/d0ra10742e PMC8695941

[28] Cenci L， Anesi A， Busato M， et al . J Mol Recognit， 2016， 29（1）： 41 26373625 10.1002/jmr.2494

[29] Saczek J， Jamieson O， Mcclements J， et al . Biosens Bioelectron， 2025， 282： 117467 40252374 10.1016/j.bios.2025.117467

[30] Ma Y-Q， Zhang S-H， Fan L-Y， et al . Journal of Northeast Normal University （Natural Science Edition）， 2006， 38（2）： 114

[31] Osman B， Uzun L， Besirli N， et al . Mater Sci Eng C， 2013， 33（7）： 3609 10.1016/j.msec.2013.04.041 23910256

[32] Moreira F T C， Sharma S， Dutra R A F， et al . Sensors and Actuators B-Chemical， 2014， 196： 123

[33] Piloto A M， Ribeiro D S M， Rodrigues S S M， et al . Sci Rep， 2018， 8（1）： 4944 29563532 10.1038/s41598-018-23271-z PMC5862838

[34] Wang H， Liu S， Xing Y-L， et al . Chinese Critical Care Medicine， 2011， 23（12）： 723 22153008

[35] Chen Y W， Rick J， Chou T C . Org Biomol Chem， 2009， 7（3）： 488 19156314 10.1039/b813361a

[36] Tai D F， Ho Y F， Wu C H， et al . Analyst， 2011， 136（11）： 2230 21519590 10.1039/c0an00919a

[37] Wang C Y， Chen Y C， Sheu D C， et al . J Taiwan Inst Chem Eng， 2012， 43（2）： 188

[38] Li Y， Yang H H， Zhuang Z X， et al . Chemical Journal of Chinese Universities-Chinese， 2005， 26（9）： 1634

[39] Lamaoui A， Amine A . Curr Top Med Chem， 2022， 22（7）： 529 35255794 10.2174/1568026622666220307124003

[40] Tang W T， Han J L， Zhang W H， et al . Analyst， 2024， 149（23）： 5617 39508117 10.1039/d4an01103a

[41] Wu D， Chen S M， Xu W H， et al . Journal of Instrumental Analysis， 2025， 44（5）： 947

[42] Liu M， Tang S S， Wang Y W， et al . Microchem J， 2024， 200： 110376

[43] Zhang Y， Cui Y Y， Sun M M， et al . Biosens Bioelectron， 2022， 209： 114262 35429772 10.1016/j.bios.2022.114262

[44] Li J， Zhang S， Zhang L， et al . Front Chem， 2021， 9： 680593 34055747 10.3389/fchem.2021.680593 PMC8162784

[45] Alahmad W， Budak F， Kaya S I， et al . Microchem J， 2025， 211： 113094

[46] Ting W T， Wang M J， Howlader M M R . Sens Actuators B Chem， 2024， 404： 135282

[47] Zuo J， Zhao X， Ju X， et al . Electroanalysis， 2016， 28（9）： 2044

[48] Ma Y， Shen X L， Wang H S， et al . Anal Biochem， 2017， 520： 9 28024754 10.1016/j.ab.2016.12.018

[49] He S， Zhang P， Sun J， et al . Biosens Bioelectron， 2022， 201： 113962 35021132 10.1016/j.bios.2022.113962

[50] Moreira F T C， Dutra R A F， Noronha J P C， et al . Electrochimica Acta， 2013， 107： 481

[51] Sadeghi Googheri M S， Campagnol D， Ugo P， et al . Chemosensors， 2025，13（1）： 26

[52] Ahmed S S， Youssef A O， Mohamed E H， et al . Spectrochim Acta A， 2023， 300： 122887 10.1016/j.saa.2023.122887 37224630

[53] Chen X， Wen Y H， Zhou N， et al . Chinese Science Bulletin， 2024， 69（11）： 1429

[54] Bai T， Wang M， Cao M， et al . Anal Bioanal Chem， 2018， 410（9）： 2291 29445833 10.1007/s00216-018-0850-z

[55] Wang X F， Zhang Y， Gu J Y， et al . Pharmaceutical Practice and Service， 2025， 43（5）： 205

[56] Jiang L . ［PhD Dissertation］. Hangzhou： Zhejiang University， 2018

[57] Palladino P， Minunni M， Scarano S . Biosens Bioelectron， 2018， 106： 93 29414095 10.1016/j.bios.2018.01.068

[58] Baldoneschi V， Palladino P， Banchini M， et al . Biosens Bioelectron， 2020， 157： 112161 32250934 10.1016/j.bios.2020.112161

[59] Piloto A M L， Ribeiro D S M， Rodrigues S S M， et al . ACS Appl Bio Mater， 2021， 4（5）： 4224 10.1021/acsabm.1c00039 35006835

[60] Zhang H Q， Hu C J， Qi P J， et al . Journal of Biomedical Engineering， 2025， 42（1）： 205 40000194 10.7507/1001-5515.202406061 PMC11955327

[61] Zadeh Z B， Hosseini S M， Mohammadnejad J， et al . ACS Appl Bio Mater， 2023， 6（7）： 2622 10.1021/acsabm.3c00257 37338424

[62] Yuan J K， Cao W Y， Zhou Y L， et al . Chinese Journal of Veterinary Science， 2024， 44（6）： 1342

[63] Alahmad W， Cetinkaya A， Kaya S I， et al . TrAC-Trends Anal Chem， 2024， 170： 117475

[64] Lim W Y， Goh C-H， Thevarajah T M， et al . Biosens Bioelectron， 2020， 147： 111792 31678828 10.1016/j.bios.2019.111792

[65] Khachornsakkul K， Trakoolwilaiwan T， Del-Rio-Ruiz R， et al . ACS Sens， 2025， 10： 5008 40591479 10.1021/acssensors.5c00940

[66] Cetinkaya A， Kaya S I， Ozkan S A . Anal Chim Acta， 2025， 1357： 344080 40316385 10.1016/j.aca.2025.344080

[67] Li W P， Xiang J W， Han J L， et al . Analyst， 2023， 148（23）： 5896 37847494 10.1039/d3an01367g

[68] Mahmarian J J， Pratt C M . J Nucl Cardiol， 2007， 14： 282 17556161 10.1016/j.nuclcard.2007.04.007

[69] Kottwitz J， Bruno K A， Berg J， et al . J Cardiovasc Transl Res， 2020， 13（5）： 853 32006209 10.1007/s12265-020-09957-8 PMC7541375

[70] ALGani F A . Int J Biol Med Res， 2011， 2（3）： 762

[71] Hasabnis G K， Altintas Z . ACS Omega， 2024， 9（28）： 30737 39035901 10.1021/acsomega.4c03252 PMC11256321

[72] Zhang G， Zhang L， Yu Y， et al . Biosens Bioelectron， 2020， 167： 112502 32836089 10.1016/j.bios.2020.112502

[73] Choudhary S， Altintas Z . Biosensors-Basel， 2023， 13（2）： 229 36831995 10.3390/bios13020229 PMC9953663

[74] Wang S， Qin J， Liang Y， et al . Anal Chim Acta， 2024， 1332： 343316 39580185 10.1016/j.aca.2024.343316

